# Incidence and Trends of Blastomycosis-Associated Hospitalizations in the United States

**DOI:** 10.1371/journal.pone.0105466

**Published:** 2014-08-15

**Authors:** Amy E. Seitz, Naji Younes, Claudia A. Steiner, D. Rebecca Prevots

**Affiliations:** 1 Epidemiology Unit, Laboratory of Clinical Infectious Diseases, National Institute of Allergy and Infectious Diseases, National Institutes of Health, Bethesda, Maryland, United States of America; 2 Department of Epidemiology and Biostatistics, Milken Institute School of Public Health, The George Washington University, Washington, D.C., United States of America; 3 Healthcare Cost Utilization Project, United States Agency for Healthcare Research and Quality, Rockville, Maryland, United States of America; California Department of Public Health, United States of America

## Abstract

We used the State Inpatient Databases from the United States Agency for Healthcare Research and Quality to provide state-specific age-adjusted blastomycosis-associated hospitalization incidence throughout the entire United States. Among the 46 states studied, states within the Mississippi and Ohio River valleys had the highest age-adjusted hospitalization incidence. Specifically, Wisconsin had the highest age-adjusted hospitalization incidence (2.9 hospitalizations per 100,000 person-years). Trends were studied in the five highest hospitalization incidence states. From 2000 to 2011, blastomycosis-associated hospitalizations increased significantly in Illinois and Kentucky with an average annual increase of 4.4% and 8.4%, respectively. Trends varied significantly by state. Overall, 64% of blastomycosis-associated hospitalizations were among men and the median age at hospitalization was 53 years. This analysis provides a complete epidemiologic description of blastomycosis-associated hospitalizations throughout the endemic area in the United States.

## Introduction

*Blastomyces dermatitidis* and the newly described *Blastomyces gilchristi* [1], dimorphic fungi, are capable of causing blastomycosis infection among humans, dogs, and other mammals. The primary route of infection is through inhalation of fungal conidia [2]. Severity of infection in humans can vary greatly with the most severe cases resulting in death. The epidemiologic features are poorly understood as the organism is difficult to culture from the environment [3] and disease reporting is only mandatory in limited states within the endemic area (AR, WI, MN, MS, LA, MI). Illinois ended mandatory reporting in 2008 [4], [5]. The organism exists in areas surrounding the Ohio and Mississippi River Valleys and the Great Lakes, and southern parts of Canada [5]–[8]. The area of the endemic region is substantially greater than that of the six US states currently with mandatory reporting.

Diagnosis is most accurately achieved through culture from a clinical specimen and initial results may be obtained as early as after five days of incubation if clinical suspicion is high [9]. However, final confirmation by culture may take up to 30 days [9]. Serological diagnosis is also possible but some tests are limited by low sensitivity and a high degree of cross reactivity, especially with histoplasmosis [9]–[11]. This is a particular problem given the overlap in endemic regions for these two organisms [8]. Unlike other mycoses, blastomycosis is only rarely considered an opportunistic infection [12]. Diagnostic delays can increase morbidity and mortality [2].

Blastomycosis occurs sporadically and in outbreaks. Outbreaks have been reported in multiple states with and without mandatory reporting [7], [13]–[16]. A recent large outbreak in Wisconsin during 2010 [13] highlighted the need for an updated epidemiologic assessment of this disease. Additionally, other reports throughout the endemic area are either outdated [17]–[19], describe local areas [14], [20], [21], specific populations [8] or outbreaks only [13], [22], [23].

Some previous epidemiologic reports suggest the possibility of an increasing incidence of blastomycosis cases [2], [5], [13], [24]. Additionally, recently reported incidences are frequently higher than those reported in previous decades for the same geographic area [8], [17]–[19], [25]. However, to our knowledge, no studies have assessed the possibility of an increasing trend in multiple states in the endemic area.

An updated and comprehensive description of national incidence and trends for blastomycosis is essential for improving the public health response to blastomycosis and highlighting geographic regions where blastomycosis should be considered in a differential diagnosis for patients that resided or traveled to these areas. This study uses the State Inpatient Database from the US Agency for Healthcare Research and Quality to provide updated and nationally representative data on blastomycosis epidemiology.

## Methods

We used the intramural State Inpatient Database (SID) from the US Agency for Healthcare Research and Quality (AHRQ), through an established collaborative effort, to describe age-adjusted state-specific blastomycosis-associated hospitalization incidence and trends. The AHRQ SID are produced as part of the Healthcare Cost and Utilization Project (HCUP) and include inpatient records from community hospitals and some non-community hosptials in each participating state [26]. According to the American Hospital Association, community hospitals make up 87% of all US registered hospitals and 95% of total US admissions [27]. They include nonfederal, short-term general, academic and teaching hospitals [27]. Hospitalization records were identified and extracted from the AHRQ SID database using the International Classification of Diseases, 9^th^ Revision, Clinical Modification codes (ICD-9-CM). A blastomycosis-associated hospitalization was defined as a hospital admission with any (primary or secondary) diagnosis of blastomycosis (ICD-9-CM: 116.0 and 116). For comparison, we also collected all records with an ICD-9-CM code for histoplasmosis (115.xx). For each hospitalization, we collected age, race, sex, state of hospitalization, patient state of residence and ICD-9-CM diagnostic codes for concurrent conditions. We extracted Current Procedural Terminology (CPT) codes for a subset of states in the endemic area to provide information regarding the diagnostic practices for blastomycosis in these states.

We included hospitalization records for all states with at least partial participation (at least one year) in the intramural SID from 2007 to 2011 for calculating blastomycosis-associated hospitalization incidence; 46 states were included in this analysis (Table 1), representing 97% of the total US population in 2010. Further, to assess trends, we extracted hospitalization records starting in 2000 or the most recent years available for the five states identified as having the highest hospitalization incidence.

**Table 1:** Years and states included in analysis.

States	Years included in analysis
AZ, AR, CA, CO, CT, FL, GA, HI, IL, IN, IA, KS, KY, ME, MD, MA, MI, MN, MO, NE, NV, NH, NJ, NY, NC, OH, OK, OR, RI, SC, SD, TN, TX, UT, VT, VA, WA, WV, WI, WY	2007–2011
AK, MS	2010–2011
LA, PA	2008–2011
MT, NM	2009–2011
DE, ND, ID, AL, DC	Not included

State-specific hospitalization incidence was calculated as the average annual number of blastomycosis-associated hospitalizations in the state divided by total person-years for that state in 2010. The 2010 state-specific census data was used to approximate the total person-years contributed by each state in 2010. The state-specific incidence was age adjusted to the 2010 United States population using the direct method of standardization as described by Woodward [28]. Because of the endemic and geographically focal nature of blastomycosis, we did not report an overall US hospitalization incidence and instead report only state-specific hospitalization incidence. We also calculated state-specific hospitalization rates using total number of hospitalizations in each state in 2010 as the denominator to assess potential bias.

Poisson regression was used to assess significance of trends (p<0.05). We used state-specific annual hospitalization incidence for this analysis. We accounted for underdispersion by scaling the standard errors using the Pearson Chi^2^ dispersion statistic. The Pearson Chi^2^ statistic was used to assess model fit. We assessed the potential for effect modification between year and state by using an interaction term.

All analyses were performed using SAS version 9.3 (SAS Institute Inc., Cary, NC).

This study was considered as not human subjects research by the National Institutes of Health Office of Human Subjects Research.

## Results

We identified 4688 total hospitalizations for blastomycosis for all states and all years in our study. The majority of the blastomycosis hospitalizations were for males (64%) and the median age at admission was 53 years. The patient demographic variable for race was available for 2959 (63%) hospitalizations. Of these hospitalizations with patient race information, 2038 (69%) were identified as white, 473 (16%) black, 211 (7%) Hispanic, 89 (3%) Asian or Pacific Islander, 40 (1%) Native American and 108 (4%) were identified as other. The majority (92%) of all blastomycosis-associated hosptailizations were for patients residing within the state in which they were hospitalized.

**Figure 1:**
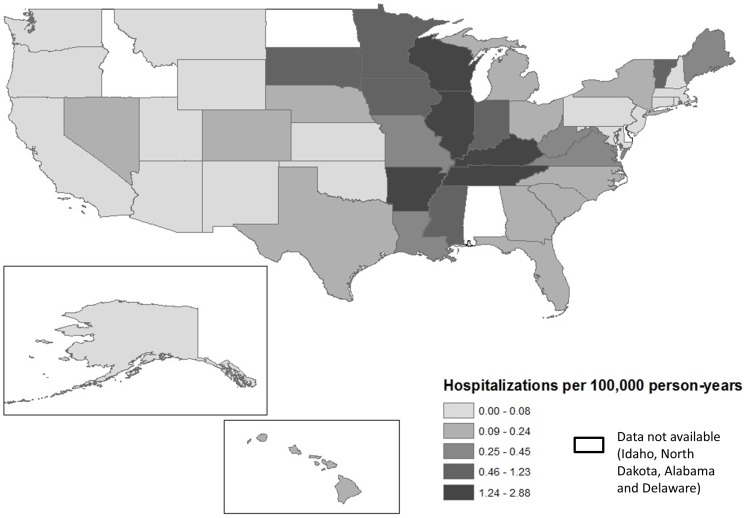
Map of age-adjusted blastomycosis-associated hospitalization incidence in the United States.

**Figure 2:**
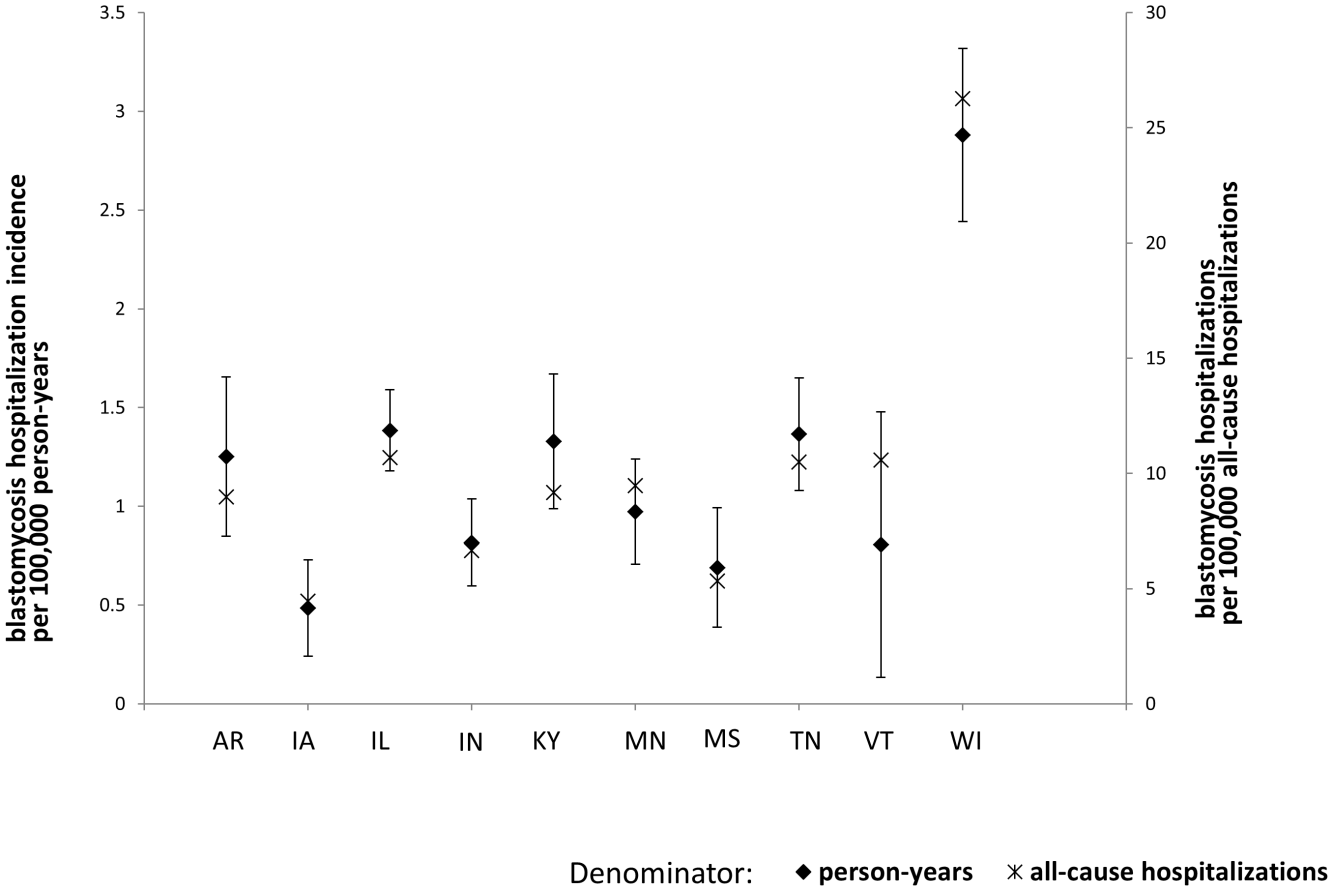
Blastomycosis-associated hospitalization incidence and rate of blastomycosis hospitalizations per all-cause hospitalization.

Blastomycosis-associated hospitalizations were most frequent in the states within the Mississippi and Ohio River valleys, with the exception of Minnesota, Iowa and Vermont (Figures 1 and 2). The state-specific age-adjusted hospitalization incidence ranged from 2.9 per 100,000 person-years in Wisconsin to less than 0.05 per 100,000 person-years in some Western and Northeastern states. Wisconsin, Illinois, Tennessee, Kentucky and Arkansas had the highest hospitalization incidence among the 46 states in our analysis, listed in order of incidence. When using total hospitalizations as the denominator, the states with the highest rates were Wisconsin, Illinois, Vermont, Tennessee and Minnesota. Although the absolute values differed, the overall pattern was similar when using either the 2010 census population or total hospitalizations as the denominator (Figure 2).

Of the 4688 hospitalizations for blastomycosis, only 247 (5.2%) had a concurrent diagnosis of HIV/AIDS or a primary immune deficiency (ICD-9-CM: 042, 279.xx). In contrast, more than 20% of histoplasmosis-associated hospitalizations had a concurrent diagnosis of HIV/AIDS or primary immune deficiency. Within the endemic area, CPT codes were only available for five states and coding within these states was incomplete; 79% of blastomycosis-associated hospitalizations were missing all CPT code information in at least one state. Thus, CPT codes were not reliable and were not included in the analysis.

**Figure 3:**
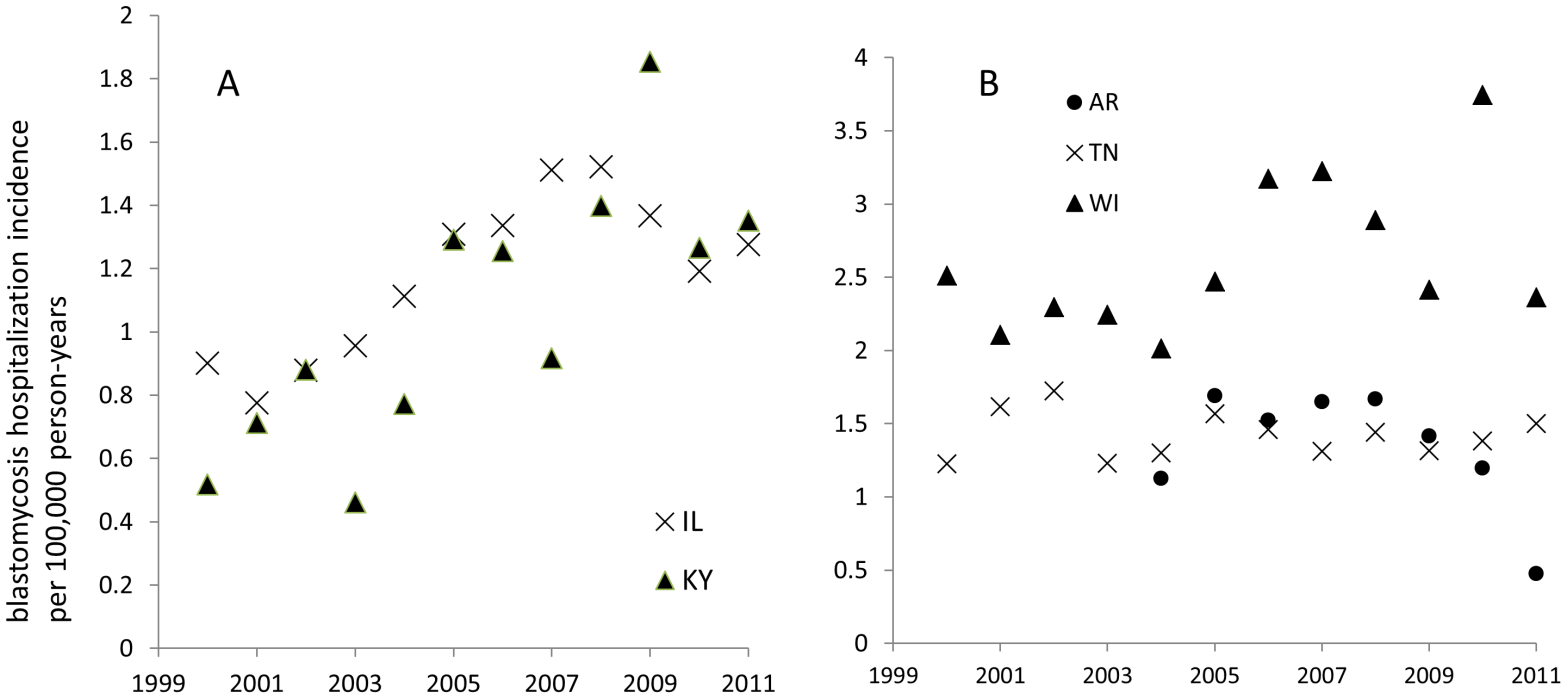
Trends in blastomycosis hospitalization incidence among states with a significant annual percentage change (A) and states with no significant annual percentage change (B).

We assessed the possibility of an increasing trend in the incidence of blastomycosis-associated hospitalizations by state for the five highest-incidence states. Illinois and Kentucky were found to have an increasing incidence of hospitalizations during the period of 2000 to 2011, with an average annual percentage change of 4.4% (95% CI: 1.8, 7.0) and 8.4% (95% CI: 3.9, 13.1) per year, respectively (Figure 3a). The 2000–2011 annual percentage changes in Wisconsin and Tennessee were not significant (WI: 2.3%, 95% CI: −5.4, 5.3; TN: −1.4%, 95% CI: −3.2, 0.53). Arkansas trend data was only available from 2004–2011 and annual percentage change during this period was also not significant (−7.2%, 95% CI: −15.2, 1.6) (Figure 3b). In the model with all states, the interaction term for state and year was found to be significant, indicating the presence of effect modification; trends varied among states.

## Discussion

In this report we update the epidemiology of blastomycosis hospitalizations in the United States. With respect to demographic features of patients with identified hospitalizations, 64% of hospitalizations were for male patients, similar to reports from prior studies of blastomycosis in an outpatient population [14], [20] and higher than what was reported for blastomycosis hospitalizations by Chu *et al* (58%) [29]. Differences in the male to female ratio may be related to the proportion of pulmonary and extra pulmonary cases with a higher proportion of women having pulmonary disease [30], [31]. The median age that we report is similar to previous reports in the outpatient population [20], [29]. The racial distribution that we observed was similar to that observed for the adult population described by Chu *et al* [29]. Only a small percentage (8%) of hospitalizations included patients residing outside of the state of hospitalization. However, we were not able to account for patients that may have traveled to an endemic area prior to their hospitalization.

We found that the current geographic distribution of hospitalization incidence is similar to what has been previously described for the endemic area of *Blastomyces dermatitidis*, with the highest incidence of blastomycosis-hospitalizations found in the states within the Mississippi and Ohio River valleys. We found a high incidence of blastomycosis-associated hospitalizations in some areas where blastomycosis is not a reportable disease; three of the five highest incidence states do not currently require blastomycosis reporting (TN, KY and IL). Our state-specific average annual incidences of hospitalizations were comparable to previous incidence reports for most states although some differences should be noted. Specifically, the average annual hospitalization incidence that we identified for Wisconsin (2.9 per 100,000 person-years) was higher than the average annual incidence for Wisconsin identified from 1986–1995 in a 1996 MMWR article (1.4 per 100,000 person-years) [19]. This increased incidence may be the result of our inclusion of a large outbreak in 2010. It could also indicate an increasing trend as our hospitalization incidence was within the range reported from a Medicare population from 1999–2008 [8]. In Illinois, we found an incidence slightly higher than what was reported for cases from the Illinois Department of Health records [5] but within the range reported from a Medicare population [8]. Blastomycosis incidence for Arkansas among persons ≥65 years was discussed in detail through letters to the editor in the journal Emerging Infectious Diseases [25], [32]. Our hospitalization incidence is slightly higher than the reported incidence rates among the elderly and lower than an incidence provided for all age groups [25] as well as an incidence reported in a separate report from 1970 [17]. In contrast, our hospitalization incidence for Kentucky was higher than the report from 1970, suggesting a changing epidemiology by state [17]. Unfortunately we were unable to include Alabama as this state does not participate in the AHRQ SID because it does not collect statewide discharge data.

We found the hospitalization incidence in Vermont to be among the ten highest state-specific hospitalization incidence rates despite having an average of less than 10 blastomycosis-hospitalizations per year. The blastomycosis endemic area stretches into areas proximal to northwestern Vermont, where much of Vermont's population resides and locally acquired disease has been documented [33]. However, instability of our incidence rates may be an issue in areas of the United States with low numbers of hospitalizations, such as Vermont. Vermont has the lowest total population besides Wyoming and District of Columbia. As such, this estimate is most likely a reflection of instability due to low numbers as reflected by a wide 95% confidence interval around the estimate (0.81 (95% CI: 0.13, 1.48)).

We used the total state population for the denominator for the majority of our analysis. However, we also calculated state-specific blastomycosis-associated hospitalizations per 100,000 total hospitalizations. Not surprisingly, the absolute estimates were different using this methodology. However, the overall relative pattern of values was similar between the two different methodologies. This suggests that differences in demographic patterns among all hospitalizations are not contributing bias to the calculation of mean blastomycosis-associated hospitalization incidence when using the state population as the denominator.

The possibility of an increasing trend was confirmed for the period of 2000–2011 for two (IL and KY) of the five states with the highest incidence of hospitalization. There was an increasing trend in Wisconsin but it was not statistically significant. No increasing trends were observed for Tennessee or Arkansas. We had fewer years of historical data available for Arkansas and a longer time period may have provided a different result. Effect modification between state and year was observed, indicating different temporal patterns for these states. This observation suggests that different factors may affect either blastomycosis infections [34], [35], hospitalizations or both in different geographic areas. Possible reasons for this increase may include increasing awareness, changing environmental factors, or an increase in disease incidence.

Only 5% of the blastomycosis-associated hospitalizations that we identified had a concurrent diagnosis of HIV/AIDS or other primary immune deficiency, indicating a minimal role of blastomycosis as an opportunistic infection as is supported by the literature [12], [36], [37]. In contrast, for another endemic mycosis, histoplasmosis, over 20% of histoplasmosis-associated hospitalizations had a concurrent diagnosis of HIV/AIDS or a primary immune deficiency, consistent with increased risk of chronic pulmonary and disseminated histoplasmosis infections among immunocompromised individuals [12]. This difference between blastomycosis-associated and histoplasmosis-associated hospitalizations suggests minimal misdiagnosis of histoplasmosis as blastomycosis in the hospitalizations in our analysis.

Some limitations should be considered when interpreting our findings. First, our data describe hospitalizations and not incident cases of blastomycosis. We were not able to identify the frequency of readmissions and may be overestimating the true incidence of blastomycosis hospitalized patients if an individual is hospitalized more than once for the same illness. The AHRQ SID provides readmission data but this is limited to fewer states and years. Additionally, when comparing publically available annual state case reports (available from state websites) to AHRQ SID hospitalization counts (available from HCUPnet [38]), we found higher counts of hospitalizations as compared to reported cases. This may also indicate underreporting of the disease. Although the absolute numbers of case counts and hospitalization counts differed, differences between years within these states and differences between states followed similar patterns; states with the highest hospitalization counts also had the highest case reports and within states, years with the highest hospitalization counts also had the highest case reports. Our estimates best represent the incidence of blastomycosis hospitalizations and using these results as estimates of case incidence should be avoided. Despite this limitation, our data are valuable for describing overall patterns of blastomycosis throughout the United States, identifying states with high burdens of disease and describing annual changes in hospitalizations and burden of disease.

A second limitation related to our use of hospitalization data is our inability to determine the diagnostic method. Multiple diagnostic assays exist for blastomycosis and many have a low specificity due to cross-reactivity, particularly with histoplasmosis. Additionally, misdiagnosis may occur with tuberculosis, malignancy [11], [39], [40] or bacterial community acquired pneumonia [14], [41], among other conditions. However, this has been shown to differ by region since physicians in areas with higher incidence may have blastomycosis higher on their list of differential diagnoses [42]. Additionally, the use of ICD-9-CM code provides little information regarding clinical diagnostic decisions and ICD-9-CM codes are primarily used for billing purposes which could contribute biases [43]. CPT codes were only available for limited states and years. We extracted these CPT codes for these states but unfortunately found unreliable coding and were not able to determine method of diagnosis.

This analysis provides an updated and population-based overview of the epidemiology of blastomycosis hospitalizations in the United States using a large dataset with a standard methodology applied across states. Our results are consistent with the overall known endemic area of the etiologic agent of blastomycosis and similar to some state-specific or local area epidemiologic reports of blastomycosis cases. This study identifies Wisconsin, Illinois, Tennessee, Kentucky and Arkansas as having the highest blastomycosis hospitalization incidence and an increasing incidence in Illinois and Kentucky. Improved physician awareness in these states may improve time to diagnosis and patient outcomes.
